# CMV-specific T-cell receptor-engineered T-cell therapy as first-line treatment for CMV reactivation after haploidentical hematopoietic stem cell transplantation: a phase 2 trial

**DOI:** 10.3389/fimmu.2026.1820399

**Published:** 2026-05-14

**Authors:** Jingwen Tang, Yanan Wen, Tian Yang, Qingyang Liu, Fei Li, Lu Wang, Zhenyang Gu, Yongli Wu, Songhua Luan, Chao Ma, Yujun Wei, Kun Qian, Liping Dou, Daihong Liu

**Affiliations:** 1 State Key Laboratory of Experimental Hematology, Senior Department of Hematology, The Fifth Medical Center of Chinese PLA General Hospital, Beijing, China; 2 Department of Intensive Care Medicine, Bethune International Peace Hospital, Hebei, China; 3 Department of Hematology, The First Medical Center of Chinese PLA General Hospital, Beijing, China

**Keywords:** adoptive cell therapy, CMV, haploidentical hematopoietic stem cell transplantation, TCR, TCR-T cell

## Abstract

**Introduction:**

Cytomegalovirus (CMV) reactivation is a major cause of mortality following haploidentical hematopoietic stem cell transplantation (haplo-HSCT). The application of conventional therapies is limited by hematologic and renal toxicities (ganciclovir and foscarnet) or time-consuming preparation (CMV-specific cytotoxic T lymphocytes [CTLs]). We previously demonstrated the efficacy and safety of CMV-specific T-cell receptor-engineered T (TCR-T) cells for treating CMV reactivation post-haplo-HSCT.

**Methods:**

We conducted a phase 2 trial using the previously established highest dose to evaluate efficacy and safety as first-line therapy in a larger cohort. Patients received CMV TCR-T cell infusions (5 × 10^5^ cells/kg) upon detection of >1 × 10^3^ copies/mL CMV DNA in two consecutive tests or >1 × 10^4^ copies/mL once. A second infusion was administered when TCR-T cell expansion remained undetectable within 7 days, and CMV load remained above 1 × 10^3^ copies/mL. Salvage therapy was initiated when complete remission (CR) was not achieved after 3 weeks. The primary endpoint was the 4-week CR rate. TCR-T cells were derived from healthy donors.

**Results:**

Among 25 patients enrolled, 13 developed CMV reactivation and received TCR-T cell therapy. Twelve (12/13, 92.3%, 95% CI: 66.7%–98.6%) achieved CR by week 4. Eleven (11/12, 91.7%, 95% CI: 64.6%–98.5%) maintained CR without further antiviral therapy, with median follow-up of 1011 (range: 657–1561) days post-infusion. Two cases of grade 1 cytokine release syndrome (CRS) occurred. TCR copy number increased ≥10^4^-fold within 14 days and remained detectable for 4 months.

**Discussion:**

These findings highlight the long-term efficacy and safety of TCR-T cells as first-line therapy for CMV reactivation post-haplo-HSCT.

**Trial Registration:**

www.clinicaltrials.gov, identifier NCT05140187.

## Introduction

Haploidentical hematopoietic stem cell transplantation (haplo-HSCT) is an important curative approach for hematopoietic malignancies. However, the incidence of cytomegalovirus (CMV) reactivation following anti-thymocyte globulin (ATG)-based transplantation is relatively high and is associated with increased mortality (1). Effective management and control of CMV reactivation after HSCT are essential to prevent progression to life-threatening CMV disease. The initiation of antiviral therapy upon reaching a defined viral load threshold, which is referred to as preemptive therapy, has become a key strategy over the past two decades to prevent CMV antigenemia from progressing to CMV disease (2). This approach reduces the risk of drug resistance and limits unnecessary exposure to antivirals (3, 4). Standard management of CMV reactivation following HSCT has relied on first-line antivirals such as ganciclovir and foscarnet; however, ganciclovir can lead to myelosuppression, and foscarnet is associated with a high incidence of nephrotoxicity (5). These limitations have prompted the exploration of cellular immunotherapies. Early studies involving donor-derived CMV-specific cytotoxic T lymphocytes (CTLs) demonstrated partial efficacy in haplo-HSCT (6). However, their clinical application remains limited owing to the time-consuming manufacturing process, low success rates, and functional exhaustion following repeated *in vitro* antigen stimulation. T-cell receptor-engineered T (TCR-T) cell therapy has emerged as a promising alternative, offering strong specificity and durable immune reconstitution with potentially reduced toxicity (7).

During the exploration of TCR-T cell therapy, the novel antiviral drug maribavir, a UL97 kinase inhibitor, was approved. Although maribavir demonstrated an improved safety profile compared with that of previous therapies, Phase 3 clinical trial data revealed a high incidence of adverse gastrointestinal reactions (8, 9). Additionally, maribavir has limited clinical application in China, with insufficient long-term safety data in Asian populations. Although the approval of maribavir has provided a new therapeutic option, the development of TCR-T therapy continues to hold value for addressing antiviral resistance and failed immune restoration through antigen-specific immune reconstitution.

TCR-T therapy, a form of adoptive immunotherapy, involves the transfer of T-cell receptor (TCR) sequences capable of recognizing specific viral or tumor antigens into peripheral blood T cells, followed by reinfusion of these genetically modified T cells into patients (10). The TCR is a disulfide-linked heterodimer comprising α and β chains that recognize 8–15 amino acid peptides presented by major histocompatibility complex (MHC) molecules, which activates and initiates the differentiation of naïve T cells into functional subsets that eliminate pathogens. The α and β chains consist of variable (V), joining (J), constant (C), and diversity (D) regions. Their recombination generates a diverse set of complementarity-determining regions (CDRs), forming a functionally diverse TCR repertoire (11). CDR sequences determine the specificity of TCR-binding to the peptide-MHC (pMHC) complex, with the third CDR (CDR3) being the most variable and directly involved in peptide recognition (12). By introducing TCR αβ chain sequences specific to tumor or viral antigens via viral transduction, TCR-T cells capable of specific antigen recognition are obtained. These transduced TCR-T cells are then cultured and expanded *in vitro* to achieve the required cell numbers for therapy. Compared with CTL therapy, TCR-T cells exhibit rapid expansion and targeted lysis of virus-infected and tumor cells, with demonstrated efficacy and low toxicity. Unlike chimeric antigen receptor (CAR)-T cells, which recognize surface antigens, TCRs recognize HLA-presented peptides derived from any cellular compartment (13). Additionally, TCRs are considerably more sensitive than CARs to low concentrations of the target antigen.

In 2009, Andrea et al. (14) first demonstrated that CMV-specific TCRs could rapidly generate TCR-transgenic T cells with potent antiviral activity *in vitro*. In 2022, Liu et al. reported the first clinical trial of TCR-T therapy in haplo-HSCT recipients with relapsed/refractory CMV reactivation. TCRs targeting multiple pp65 epitopes across three major HLA-A alleles were identified from healthy donors, validating the safety and feasibility of this strategy (15). In 2024, we reported a phase I dose-finding clinical trial to assess the primary safety and efficacy of CMV-TCR-T cells as first-line preemptive therapy in patients with CMV reactivation following haplo-HSCT (16). This study included three dose levels (1×10^3^, 1×10^5^, and 5×10^5^ cells/kg). Although both higher doses demonstrated therapeutic effects and acceptable safety, the maximum tolerated dose (MTD) could not be definitively determined. Given that the highest dose (5×10^5^ cells/kg) demonstrated a faster onset of efficacy without severe toxicity, we selected this dose for further evaluation.

Based on these findings, we conducted a phase II clinical trial to evaluate the efficacy and safety of TCR-T cells as first-line preemptive therapy for CMV reactivation following haplo-HSCT in a larger cohort. To improve population coverage, we introduced two additional HLA-A-restricted epitopes and increased the sample size accordingly.

## Methods

### Study design and endpoints

In this phase 2 clinical trial, patients who underwent haplo-HSCT at the Chinese PLA General Hospital between April 2023 and April 2024 were recruited. All enrolled patients shared one of the following HLA-A types with their donors: 02:01, 11:01, 24:02, 02:06, or 02:07 (detailed inclusion and exclusion criteria are provided in **Supplementary Table 1**). This study was approved by the Ethics Committee of the Chinese PLA General Hospital and conducted in accordance with the Declaration of Helsinki. All patients provided written informed consent before enrollment. The trial is registered at www.clinicaltrials.gov (NCT05140187).

CMV DNA copy number in peripheral blood was monitored twice weekly after HSCT. Upon CMV reactivation, CMV-TCR-T cells were infused at a dose of 5 × 10^5^ cells/kg. No CMV-active antiviral agents were administered between the detection of CMV reactivation and the first TCR-T infusion; thus, no bridging antiviral therapy was given during this interval. This 1–5 day interval was primarily used for mandatory product release procedures, including quality control testing, product transportation, and pre-infusion clinical evaluation. During this period, patients were closely monitored clinically, and short-term watchful waiting was considered acceptable because patients had isolated viremia or mild CMV disease without evidence of severe organ dysfunction. A second infusion was administered when TCR expansion was not observed within seven days, CMV load remained above 1 × 10^3^ copies/mL, or clinical symptoms of CMV disease persisted. The criteria for a third infusion were identical to those for the second infusion. Each patient could receive a maximum of three TCR-T infusions. If CMV infection remained uncontrolled after three infusions, patients were transitioned to salvage antiviral therapy according to the study protocol. During the pre-emptive TCR-T treatment window, no concomitant CMV-active antiviral agents (including ganciclovir, valganciclovir, or foscarnet) were administered. CMV-TCR-T cells were used as the sole pre-emptive intervention until CR was achieved or protocol-defined salvage therapy was initiated. When CR was not achieved after three weeks of TCR-T cell therapy, ganciclovir (5 mg/kg intravenously, bid) was initiated as salvage therapy; for patients with myelosuppression, foscarnet was used as an alternative. In cases of persistent virologic non-response, earlier transition to salvage antiviral therapy was allowed at the investigator’s clinical discretion, even before the maximum of three planned infusions had been completed.

The primary endpoint was the 4-week CR rate. Secondary endpoints included time to CR, recrudescence rate, and safety.

### Concurrent cohorts

To provide a clear framework for descriptive comparisons, all haplo-HSCT recipients during the study period were stratified into four cohorts based on HLA matching and CMV reactivation status: 1) TCR-T treatment cohort (the study cohort of the clinical trial): Patients with CMV reactivation who received TCR-T cell therapy; 2) Concurrent Cohort 1: HLA-matched patients who were eligible for TCR-T therapy but did not experience CMV reactivation; 3) Concurrent Cohort 2: HLA-mismatched patients with CMV reactivation who received standard antiviral therapy; 4) Concurrent Cohort 3: HLA-mismatched patients without CMV reactivation.

During the study period, a small number of patients who met the HLA eligibility criteria and developed CMV reactivation declined the TCR-T therapy. These patients were not included in TCR-T treatment cohort.

Transplantation procedures—including donor selection, conditioning regimen, graft-versus-host disease prophylaxis, and supportive care—were consistent across all cohorts. No patients meeting the eligibility criteria were excluded or selectively assigned. Because these cohorts were non-randomized, all efficacy comparisons are exploratory and descriptive.

The primary focus of this study was on the TCR-T-treated cohort. Short-term efficacy analyses were primarily conducted by comparing the TCR-T-treated cohort with Concurrent Cohort 2. Long-term follow-up outcomes—including sustained complete remission, deaths, relapse, and complications—were reported for all four cohorts to provide a comprehensive descriptive assessment of patient outcomes over time.

### Definitions

CMV DNA in peripheral blood was quantified in the Hematology Laboratory of the Chinese PLA General Hospital using a commercial real-time PCR assay based on the fluorescent probe method (CMV nucleic acid detection kit, SINOMD Gene, China). In our center, CMV viral load is routinely reported in copies/mL, and this unit was therefore retained throughout the manuscript to reflect the actual clinical management practice used in this study. The lower limit of detection of this assay was 1 × 10^3^ copies/mL, which is approximately equivalent to 1 × 10^3^ IU/mL, with an assay-specific conversion factor of approximately 1. For international comparability, the predefined treatment thresholds are also expressed in approximate IU/mL equivalents. CMV reactivation was defined as CMV DNA >1 × 10^3^ copies/mL in two consecutive measurements, >1 × 10^4^ copies/mL in a single measurement, or clinical symptoms with microbiological evidence of CMV disease. CMV disease was defined as clinical signs and symptoms attributable to CMV, including CMV pneumonia or gastroenteritis.

CR was defined according to the clinical presentation of CMV infection. In patients with CMV viremia, CR was defined as clearance of viremia documented by two consecutive negative CMV DNA tests, and the date of the second negative test was recorded as the time to CR. In patients with CMV disease without concurrent viremia, CR was defined as complete resolution of CMV-related symptoms or organ manifestations, and the date of clinical resolution was recorded as the time to CR. In patients with CMV disease with concurrent viremia, CR required both virological clearance and clinical resolution, and the time to CR was defined as the date on which both criteria were fulfilled (17). Ineffectiveness was defined as persistent CMV viremia or unresolved CMV disease at 3 weeks after TCR-T infusion, prompting consideration of salvage antiviral therapy according to the study protocol. Recrudescence was defined as reappearance of CMV reactivation following at least 4 weeks of CR.

For this study, the TCR-T treatment window was defined as the period from the first TCR-T infusion to achievement of CR or initiation of salvage antiviral therapy. Corticosteroid exposure at infusion was defined according to systemic corticosteroid use at the time of or immediately surrounding the first TCR-T infusion.

Cytokine release syndrome (CRS), immune effector cell-associated neurotoxicity syndrome (ICANS), and TCR-T-associated GVHD were graded according to the American Society for Transplantation and Cellular Therapy (ASTCT) criteria (18). Adverse events (AEs) were graded using the National Cancer Institute Common Terminology Criteria for Adverse Events, Version 5.0. Neutrophil engraftment was defined as the first of three consecutive days with absolute Neutrophil Count (ANC) ≥ 0.5 × 10^9^/L, and platelet engraftment as the first of seven consecutive days with platelet count ≥ 20 × 10^9^/L without transfusion support. The time to CD4+ T-cell count >50/μL was analyzed for each patient as a clinically relevant early immune recovery marker (19). OS (Overall Survival) was defined as the time from transplantation to the occurrence of death from any cause. PFS (Progression-Free Survival) was defined as the time from transplantation to the occurrence of disease progression, relapse, or death, whichever occurs first.

### Preparation of CMV-TCR-T cells

CMV TCR-T cells were prepared for all 25 patients, with detailed preparation methods provided in the **Supplementary Materials**. The median transduction efficiency of CD8+Tetramer+ cells was 28.41% (range, 11.6–77.6%), reflecting the proportion of CD8+ cells successfully expressing the TCR sequence (quality control standard: ≥1%). The total viable CD8+Tetramer+ cell count was 2.86 × 10^7^ (range, 10.0×10^6^–6.83 × 10^7^ cells). The median preparation time was 13 days (range, 10–13 days). All patients who received TCR-T products were administered at least one dose of TCR-T (5 × 10^5^ cells/kg). To better reflect antigen-specific effective cell exposure, we further summarized product viability, CD8+Tetramer+ transduction efficiency, and the estimated infused CD8+Tetramer+ cell dose for each patient (**Supplementary Table 4**). Prior to infusion, each CMV-TCR-T cell product underwent release testing including cell viability, CD3+/CD8+Tetramer+ transduction efficiency, sterility, endotoxin, and mycoplasma testing. Detailed release-related characteristics are summarized in **Supplementary Table 4**. Functional characterization further confirmed the antigen-specific activity of the engineered TCR-T cells. CMV-TCR-T cells demonstrated efficient cytotoxicity against HLA-matched target cells but not HLA-mismatched controls (**Supplementary Figure 2**).

### qPCR, immune repertoire sequencing, and immune reconstitution surveillance

Quantitative polymerase chain reaction (qPCR) was performed to monitor the amplification and persistence of CMV TCR-T *in vivo*. Peripheral blood DNA was extracted at the following time points post-infusion: days 0, 7, 11, 14, 21 ± 2 days, 28 ± 2 days, 42 ± 4 days, 56 ± 4 days, 70 ± 4 days, 114 ± 7 days, and 228 ± 7 days. DNA was extracted using the QIAamp DNA Mini Kit (QIAGEN, Germany). Quantitative real-time PCR was performed to assess CMV-TCR-T gene levels, with negativity defined as < 100 copies/µg of DNA. To monitor persistence and clonal diversity, DNA extracted from peripheral blood mononuclear cells (PBMCs) of three participants was used for immune repertoire sequencing.

Flow cytometry was used to detect lymphocyte subsets according to WS/T 360—2024 standard operating procedures (20). Peripheral blood samples were collected in EDTA anticoagulant tubes, promptly processed, and analyzed at 23 °C. Specific monoclonal antibodies were used to label T lymphocytes (including CD4+ and CD8+ T cells) and B lymphocytes (BD, USA), which were analyzed using flow cytometry (FACSCanto II, BD, USA). Four- or six-color antibody combinations were used to ensure accurate identification and quantification of lymphocyte subsets. All samples were analyzed using a performance-verified flow cytometer, with quality assured through in-house and external quality assessments.

### Transplantation procedure

The haplo-HSCT protocol included conditioning, mobilization, stem cell collection, and GVHD prophylaxis. Patients received a myeloablative regimen (Bu/Cy + ATG) consisting of cytosine arabinoside (Ara-C; 4 g/m^2^/day, days −10 and −9), busulfan (3.2 mg/kg/day, days −8 to −6), cyclophosphamide (1.8 g/m^2^/day, days −5 and −4), and anti-thymocyte globulin (ATG, 2.5 mg/kg/day, days −5 to −2) (21–23). All patients received a GVHD prophylactic regimen of short-term methotrexate (MTX, intravenously, day +1, 15mg/m^2^; day +3, 10mg/m^2^; day +6, 10mg/m^2^). Acute GVHD (aGVHD) was treated as previously described (16). CMV prophylaxis consisted of ganciclovir (50 mg/day, days −10 to −3). Acyclovir (400 mg, bid, days 1–180) was prophylactically administered against herpes simplex and varicella zoster viruses. Standard treatment for CMV reactivation involved first-line therapy with foscarnet (5 mg/kg, intravenously, twice daily). Second-line therapy consisted of combination therapy or monotherapy with ganciclovir (60 mg/kg, intravenously, bid).

CMV DNA in peripheral blood was monitored using real-time qPCR twice weekly from neutrophil engraftment to +1 month, weekly from +2 to +3 months, and biweekly from +4 to +6 months post-HSCT. The total duration of monitoring exceeded one year following transplantation. After patients achieved CR, they were followed up continuously for one week, during which CMV monitoring was performed twice. If CMV DNA remained persistently negative throughout this one-week period, we gradually extended the monitoring interval: once weekly in the first three months after CR, and once every two weeks in the 4–6 months after CR. The monitoring modality was consistent with that of routine post-transplantation surveillance. Detection of CMV viremia triggered intensified monitoring (twice weekly) to enable rapid therapeutic response evaluation and treatment adjustments. Re-treatment was initiated when any of the following criteria were met: CMV DNA > 1 × 10^3^ copies/mL on two consecutive tests, CMV DNA > 1 × 10^4^ copies/mL on a single test, or the presence of clinical symptoms with microbiological evidence of CMV infection.

### Sample size calculation

The sample size was calculated according to the CR rate of the study. Several clinical trials reported that the CR rate of patients with CMV viremia treated with ganciclovir (GCV) was 56.0% to 91.4% (24–26). CMV disease following HSCT is characterized by a mortality rate of 20%–60% (27). Given that this study targets both CMV viremia and CMV disease following allo-HSCT, we estimated the efficacy of first-line ganciclovir therapy to be approximately 45%. Based on our Phase I clinical trial, the CR for the TCR-T group was estimated to be 90% (16). A sample size of 13 achieves 80% power to detect differences using a two-sided exact test with a significance level (alpha) of 0.05 (PASS Software, NCSS LCC, USA). Allowing for a CMV reactivation rate of 60% to 83% (28, 29), about 16–22 patients were required.

### Statistical analysis

All analyses were performed using Prism Software (version 10.2.0; GraphPad Software Inc., Boston, MA, USA) or R Version 4.1.2 (www.cran.r-project.org). Overall survival (OS) and progression-free survival (PFS) were estimated using the Kaplan–Meier method. The cumulative incidence rates of NRM and relapse were estimated in the competing risk framework, with each being treated as a competing event. The cumulative incidence of aGVHD was also estimated in the competing risk framework, with relapse or death without developing GVHD as a competing event. All p values were two-sided at the significance level of 0.05 unless otherwise stated.

## Results

### Patient characteristics

**Figure 1:**
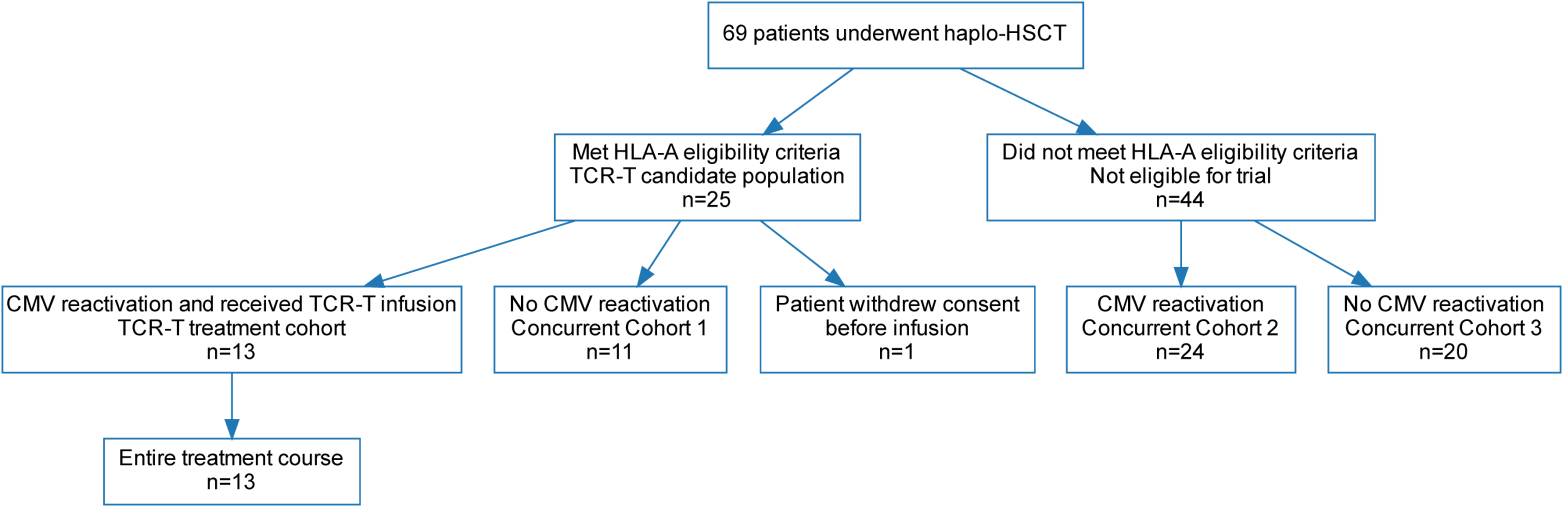
Flowchart of patient enrollment and clinical study design.

In our study, we considered five HLA-A loci commonly found in the Chinese population (HLA-A*02:01, HLA-A*24:02, HLA-A*11:01, HLA-A*02:06, and HLA-A*02:07). HLA restrictions for all TCR constructs are summarized in **Supplementary Table 5**. During the enrollment period, a total of 69 patients underwent haplo-HSCT at our center. Among them, 25 patients met the prespecified HLA-A eligibility criteria for the TCR-T study and entered the TCR-T candidate population. Donor-derived peripheral blood lymphocytes matched to the shared HLA-A allele were used to successfully generate CMV-specific TCR-T cells. Of these 25 patients, 14 developed CMV reactivation. Among them, 13 received TCR-T infusion (TCR-T treatment cohort), whereas 1 withdrew consent before infusion. Baseline characteristics are detailed in **Table 1**. The remaining 11 patients did not develop CMV reactivation (Concurrent Cohort 1). The remaining 44 patients did not meet the HLA-A matching criteria required for TCR-T therapy and were therefore not eligible for trial enrollment. Among these 44 patients, 24 developed CMV reactivation (Concurrent Cohort 2), whereas 20 did not experience CMV reactivation (Concurrent Cohort 3) (**Figure 1**). **Supplementary Table 2** summarizes the demographic, clinical, and transplantation characteristics of the TCR-T andconcurrent control cohorts.

**Table 1:** Baseline characteristics of all patients.

Patient no.	Diagnosis	Patient’s HLA-A	Donor		Donor’s HLA-A	D/R CMV serostatus	Conditioning regimen	aGVHD			
								aGvHD after infusion	Day of aGvHD	Treatment protocol	Pre-infusion aGVHD assessment
1	MDS	02:01, 02:07		Daughter Son	02:01, 02:01	D+/R+	BU/CY+ATG	Grade 2 skin and GI GVHD	20	Steroids 1mg/kg+Rux 5 mg/day	CR
2	NHL; ALL	02:03, 24:02		Son	02:03, 24:02	D+/R+	FB+ATG	No	–	–	–
3	AML	24:02, 30:01		Daughter	24:02, 02:01	D+/R+	FB+ATG	No	–	–	–
4	AML	02:01, 02:01		Sibling	02:01, 02:06	D+/R+	FB+ATG	Grade 3 GI GVHD	77	Steroids 1mg/kg+basiliximab 5 doses	PR
5	AML	11:01, 02:07		Sibling	11:01, 11:01	D+/R+	BU/CY+ATG	No	–	–	–
6	AML	02:07, 24:02		Son	02:07, 24:02	D+/R+	FB+ATG	No	–	–	–
7	ALL	24:02, 03:01		Daughter	24:02, 02:07	D+/R+	FB+ATG	No	–	–	–
8	AML	02:01, 24:02		Sibling	02:01, 26:01	D+/R+	FB+ATG	No	–	–	–
9	AML	24:02, 02:06		Father	24:02, 33:03	D+/R+	BU/CY+ATG	Grade 3 skin GVHD	30	Steroids 1mg/kg+Rux 5 mg/day	CR (steroid reduction stage: 44mg/day)
10	ALL	11:01, 24:04		Daughter	11:01, 24:02	D+/R+	FB+ATG	No	–	–	–
11	AML	24:02, 02:06		Mother	11:01, 02:06	D+/R+	FB+ATG	Grade 2 skin and GI GVHD	26	Steroids 1mg/kg	CR (steroids discontinued)
12	AML	02:01, 24:02		Son	02:01, 24:02	D+/R+	BU/CY+ATG	Grade 2 skin and GI GVHD	17	Steroids 1mg/kg+Rux 5 mg/day+basiliximab 3 doses	PR (steroids discontinued)
13	AML	11:01, 02:06		Son	11:01, 02:06	D+/R+	BU/CY+ATG	No	–	–	–

**Figure 2:**
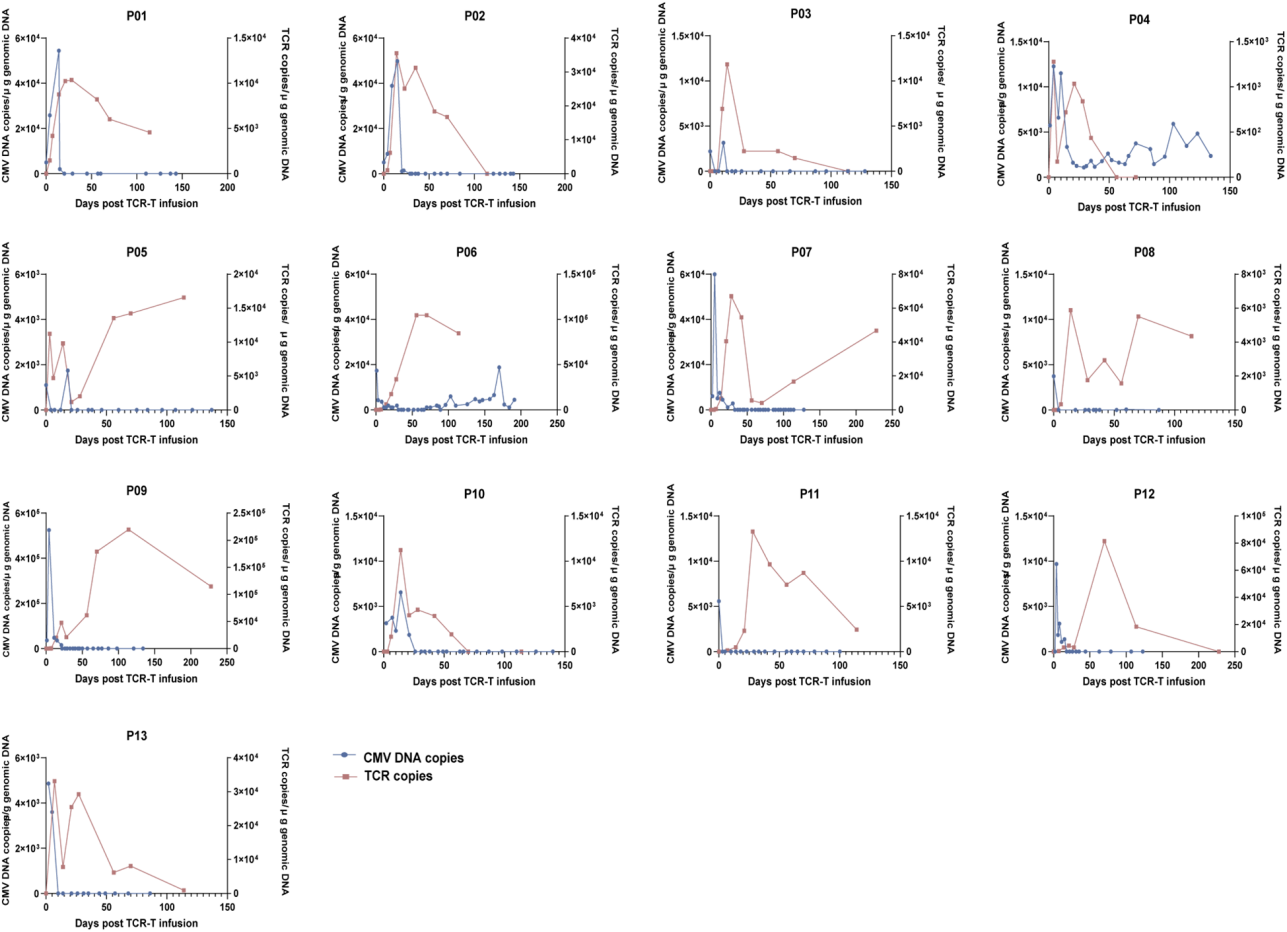
Dynamics of viral load (left y-axis) and TCR copy number (right y-axis) in individual patients following TCR-T cell infusion.

Thirteen patients (13/25, 52.0%) developed CMV reactivation and received CMV-TCR-T cell infusion as first-line preemptive therapy (**Figure 2**). Clinical presentations included isolated viremia (defined as detectable CMV DNA measurement with no evidence of organ involvement) in 10 patients (10/13, 76.9%) and CMV disease in three patients (3/13, 23.1%), comprising two cases of colitis and one of probable pneumonia. The CMV viral load at the time of treatment trigger for each treated patient is summarized in **Table 2**. Among the five high-risk patients who had developed aGVHD before TCR-T therapy and were still receiving tapering steroids or other ongoing immunosuppressive therapies at the time of CMV reactivation (Patients 01, 04, 09, 11, and 12), none had detectable but subthreshold viremia prior to meeting the prespecified trigger criteria. The median time to CMV reactivation was 51 days post-HSCT (range, 30–83 days), and the median time to TCR-T infusion was 3 days (range, 1–5 days) following reactivation.

**Table 2:** Outcomes of all patients treated with TCR-T cells.

Patient ID	Day of CMV reactivation	Time to neutrophil engraftment	Time to platelet engraftment	Time to CD4^+^ T cells >50/μL	CMV reactivation symptom	CMV viral load at the time of treatment	Peak CMV viral load	Day of TCR-T cell infusion post-HSCT	Response to first-line therapy	Time to achieve CR (days)	Recrudescence	Concomitant CMV-active antiviral during TCR-T treatment window	Salvage / subsequent antiviral therapy
01	36	12	9	125	Viremia	4960	54400	41	CR	21	No	None	None
02	65	11	10	131	Viremia	2206	49800	66, 72	CR	22	No	None	None
03	52	15	15	299	Viremia	5013	3160	56	CR	14	No	None	None
04	83	12	11	135	Colitis	5730	12270	85, 92	Ineffective	–	No	None	Ganciclovir
05	43	10	11	80	Viremia	1100	1740	45, 60	CR	4	No	None	None
06	51	15	14	83	Viremia	17300	17300	54, 61	CR	17	Yes	None	Ganciclovir + foscarnet
07	37	16	25	110	Colitis	6000	59950	40, 46, 52	CR	22	No	None	None
08	61	15	15	99	Viremia	3730	3730	63	CR	18	No	None	None
09	37	11	9	71	Pneumonia	36200	524800	40	CR	22	No	None	None
10	46	12	13	88	Viremia	3140	6552	47	CR	21	No	None	None
11	60	12	14	94	Viremia	5580	5580	64	CR	4	No	None	None
12	30	10	10	77	Viremia	9696	9696	33, 39, 46	CR	22	No	None	None
13	54	14	25	95	Viremia	4862	4862	57	CR	4	No	None	None

Five of the 13 patients (Patients 01, 04, 09, 11, and 12) developed aGVHD before CMV reactivation and received steroids, ruxolitinib, and basiliximab for management.

### High remission and viral clearance achieved with TCR-T cell therapy

For the 13 patients with CMV reactivation, the median follow-up time was 1011 days (range, 657–1561 days) post-HSCT and 812 days (range, 607–1086 days) post-infusion. Eight patients (8/13, 61.5%, 95% CI: 35.5%–82.3%) achieved CR within 3 weeks; twelve patients (12/13, 92.3%, 95% CI: 66.7%–98.6%) achieved CR within 4 weeks. No patient received concomitant CMV-active antiviral therapy during the TCR-T treatment window; antiviral agents were introduced only as salvage therapy in patients who failed to achieve protocol-defined CR or after subsequent recrudescence. Eleven patients maintained sustained CR throughout follow-up without additional antiviral therapy (**Figure 2**). One patient (Patient 06) experienced recrudescence on day 104 post-HSCT, 33 days after achieving CR, corresponding to 1 of 12 patients who achieved CR (1/12, 8.3%). One patient (Patient 04) did not respond. Because CMV DNA remained detectable after two TCR-T infusions, salvage therapy with ganciclovir was initiated on day 99 post-HSCT; however, CMV DNA remained persistently detectable. The median time to first infusion was 50 days (range, 30–120 days) post-HSCT, and the median time to CR was 22 days (range, 4–29 days) post-infusion (**Table 1**).

Three out of 13 patients developed CMV disease. Patient 09 presented with persistently elevated CMV DNA from day 36 post-HSCT. Fever, cough, and dyspnea developed on day 43. Bacterial, fungal, and other respiratory viral infections were comprehensively excluded by routine microbiological examinations. CMV DNA was detected by real-time quantitative PCR in bronchoalveolar lavage fluid, suggesting probable CMV pneumonia. TCR-T infusion was administered on day 39 post-HSCT. Five days post-infusion, body temperature normalized, oxygenation index increased to 400 mmHg, and respiratory symptoms resolved. CMV DNA became undetecTable 20 days post-TCR-T infusion and remained negative during follow-up. Patient 07 developed CMV reactivation on day 37 post-HSCT, presenting with colitis characterized by left lower abdominal pain, diarrhea (13–15 times/day), tenesmus, and gross bloody stools. CMV inclusion bodies were confirmed via colonoscopic biopsy. CMV was detected in peripheral blood on day 40. After three TCR-T infusions (days 40, 46, and 52), diarrhea frequency decreased to ≤3 times/day, stool consistency returned to normal, CMV DNA was undetectable, and no CMV reactivation occurred during follow-up.

Patient 04 developed a high fever (39.2 °C) and watery diarrhea (8–10 times/day) on day 83 post-HSCT. Although CMV DNA was initially undetectable in peripheral blood, CMV inclusion bodies were identified on colonic biopsy, and peripheral blood CMV DNA became detectable three days later. Two TCR-T infusions were administered on days 85 and 92. Nine days after the first infusion, clinical symptoms improved substantially, with resolution of fever and normalization of bowel movements (1–2 semi-formed stools/day). Follow-up colonoscopic pathology also showed resolution of acute inflammation and disappearance of CMV inclusion bodies. Because peripheral blood CMV DNA remained detectable after two TCR-T infusions and protocol-defined CR had not been achieved, salvage therapy with ganciclovir was initiated on day 99 post-HSCT, before the formal 3-week assessment time point calculated from the first infusion, owing to persistent virologic nonresponse. CMV DNA nevertheless remained persistently detectable thereafter. The patient died on day 154 from severe bacterial infection and multi-organ failure.

**Figure 3:**
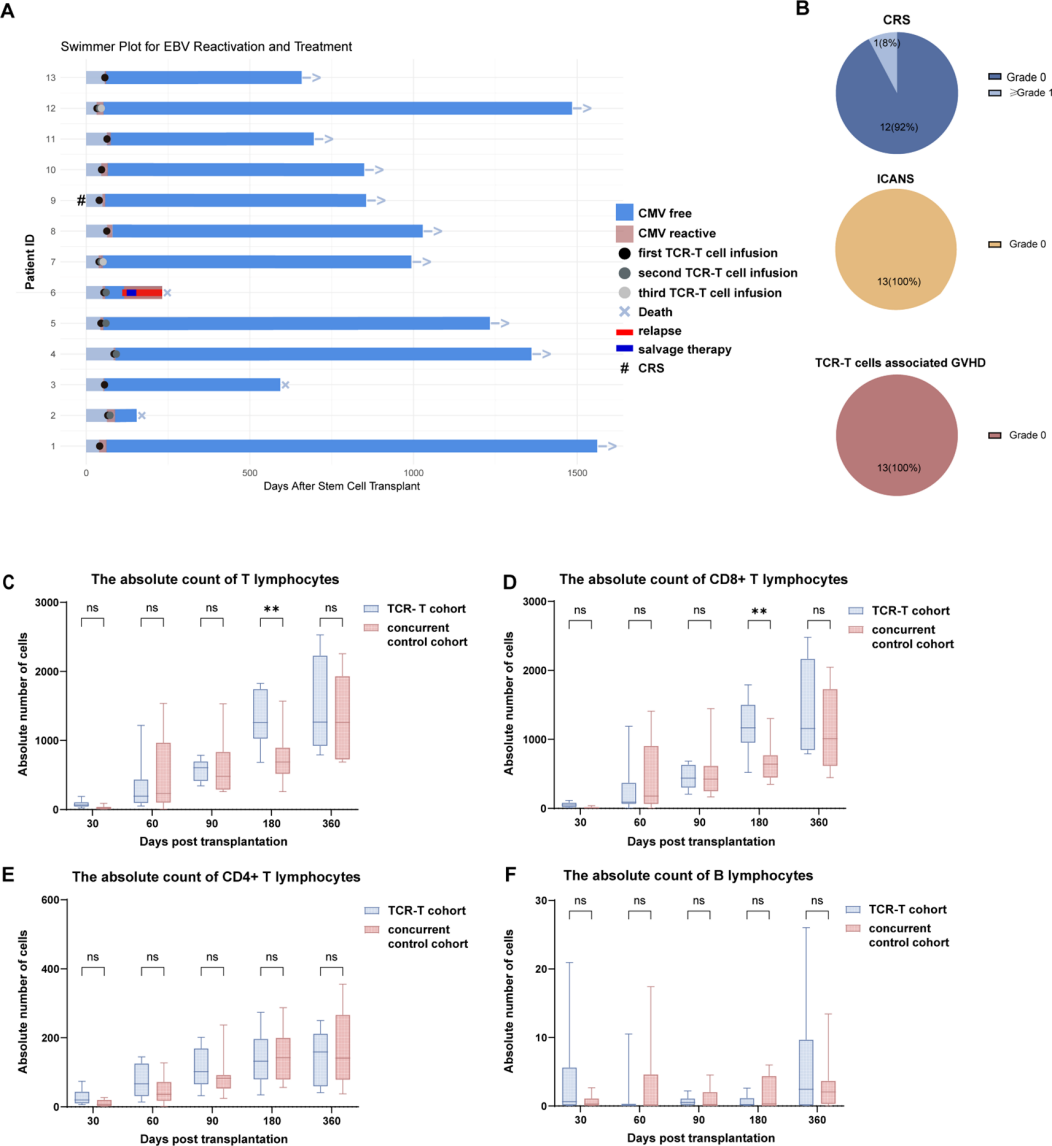
Clinical responses of patients. **(A)** Timeline of treatment and follow-up events. **(B)** Statistical analysis of potential adverse events during the treatment course. CRS: cytokine release syndrome; ICANS: immune effector cell-associated neurotoxicity syndrome; GVHD: graft-versus-host disease. **(C)** Dynamics of absolute T-cell counts (cells/mL) in peripheral blood. **(D)** CD8+ T-cell counts (cells/mL) in peripheral blood. **(E)** CD4+ T-cell counts (cells/mL) in peripheral blood. **(F)** B-cell counts (cells/mL) in peripheral blood. **, p < 0.01; ns, not significant.

Among the 13 patients with CMV reactivation, one case of CMV recrudescence was recorded. Patient 06 developed CMV reactivation on day 51 post-HSCT, with CMV DNA copy numbers exceeding 1 × 10^4^ copies/mL. Two TCR-T cell infusion doses were administered (days 54 and 67), and CR was achieved on day 71 and sustained for nearly 5 weeks. Leukemia relapse occurred on day 95 post-HSCT, followed by CMV recrudescence on day 104. Despite salvage therapy with ganciclovir and foscarnet, viremia persisted without remission. The patient died of leukemia relapse on day 232 post-HSCT (**Figure 3A**).

### CMV-TCR-T cell therapy was safe and well-tolerated

CMV-TCR-T cell treatment was well-tolerated. No clinically significant allergic reactions occurred within 30 minutes to 3 hours post-infusion. By the last follow-up (February 15, 2025), no serious adverse events (SAEs), ICANS, TCR-T cell-associated GVHD, or secondary tumors were observed in any of the patients. Patients 03 and 11 developed grade 1 CRS on the infusion day, each presenting with fever. Patient 09 had a 1-day fever peak of 39.3 °C, and Patient 11 had a peak of 38.4 °C lasting under 2 days. No grade II–IV CRS or treatment-related fatalities occurred. All these patients became afebrile without the need for antipyretic therapy, and no other adverse effects were observed (**Figure 3B**).

### TCR-T therapy did not impair immune reconstitution

All patients in the CMV-TCR-T cohort achieved neutrophil and platelet engraftment prior to TCR-T cell infusion, which ruled out the possibility of primary graft failure. Notably, CMV reactivation in all treated patients occurred after engraftment. We further analyzed the time to CD4^+^ T-cell count >50/μL in each patient, a well-validated optimal biomarker for early immune recovery in various HSCT settings (1). Among the responding patients, except for Patient 04, all achieved CD4^+^ T-cell count >50/μL after TCR-T cell infusion and the subsequent achievement of complete remission (CR). This indicated that the endogenous CMV-specific immunity of these responding patients had not yet been restored at the time of TCR-T treatment, confirming that viral clearance was not solely due to pre-existing immune recovery but was definitively attributed to the infused TCR-T cells. For Patient 04, the non-responder, CD4^+^ T cells reached >50/μL on day 135 post-HSCT, which was later than the time of TCR-T cell infusion. This temporal relationship demonstrated that the poor TCR-T expansion in Patient 04 could not be explained by advanced endogenous T-cell reconstitution, but was more likely associated with other factors.

Lymphocyte subset reconstitution (CD3^+^ T, CD4^+^ T, CD8^+^ T, B, and NK cells) was compared between the TCR-T and concurrent control cohorts at the same time points post-HSCT. TCR-T cells exerted minimal effects on cellular immune reconstitution (**Table 3**; **Figure 3C–F**). On day 180 post-HSCT, the TCR-T cohort exhibited significantly higher levels of CD3^+^ (p=0.02) and CD8^+^ (p=0.03) T cells than those in the control cohort. No significant differences were observed at other time points.

**Table 3:** Dynamics of lymphocyte subsets.

Days after HSCT	Group	CD3^+^ T cells (cells/μL) [IQR]	P value	CD4^+^ T cells (cells/μL) [IQR]	P value	CD8^+^ T cells (cells/μL) [IQR]	P value
30	TCR-T cohort	66.1[21.26–190.25]	>0.99	19.98[6.46–74.36]	0.97	51.1[9.46–115.89]	>0.99
	Control cohort	19.6[0.80–89.8]		7.33[0.23–27.18]		5.76[0.57–53.00]	
60	TCR-T cohort	193.1[49.6–1219.4]	0.85	66.9[14.17–144.82]	0.85	90.6[4.08–1191.18]	0.71
	Control cohort	233.2[5.1–1536.4]		36.47[1.45–127.41]		178.99[3.69–1408.98]	
90	TCR-T cohort	631.0[884.84–343.6]	0.93	101.9[32.8–201.7]	0.87	438.6[206.6–684.6]	0.97
	Control cohort	423.9[871.9–259.7]		83.0[24.3–237.3]		425.5[166.5–1444.7]	
180	TCR-T cohort	1259.8[684.2–1824.9]	0.02	125.91[34.6–273.51]	>0.99	1168.2[520.5–1790.3]	0.03
	Control cohort	819.75[258.1–1647.8]		142.36[56.5–287.3]		648.6[648.6–1301.0]	
360	TCR-T cohort	1265.7[790.6–2526.9]	0.73	159.105[41.37–250.12]	0.88	1158.3[790.55–2480.33]	0.34
	Control cohort	1261.6[687.4–2255.8]		141.3[37.72–355.84]		1011.7[447.31–2046.01]	

### Favorable long-term survival achieved with TCR-T cell therapy

Three patients (3/13, 23.1%) died: Patient 04 died of multi-organ failure secondary to severe bacterial infection (OS: 154 days), Patient 09 of influenza A infection (OS: 593 days), and Patient 06 of relapse (OS: 232 days). Notably, although both Patients 04 and 06 had uncontrolled CMV infection at death, their clinical trajectories differed substantially. Patient 04 had primary treatment failure with persistent viremia and no CR. Patient 06 initially achieved CR after TCR-T therapy, but developed CMV recrudescence following leukemia relapse; salvage antiviral therapy failed, likely due to profound immunosuppression from progressive hematologic malignancy. Reinfusion of TCR-T cells was considered but not feasible due to the patient’s poor clinical condition. All other patients remained alive at the final analysis.

**Figure 4:**
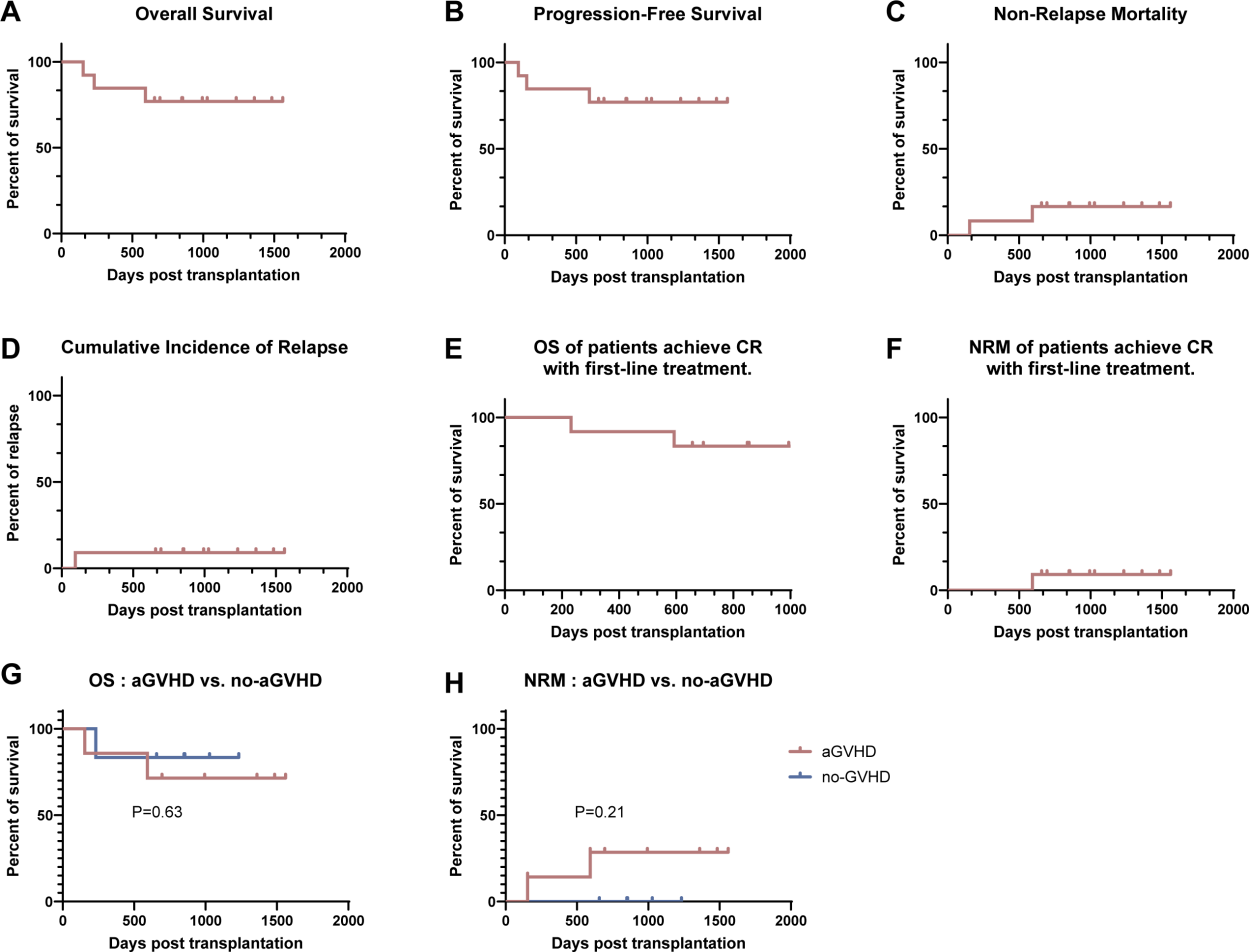
Long-term response and survival outcomes. **(A)** Overall survival **(OS)** rate. **(B)** Progression-free survival (PFS). **(C)** Non-relapse mortality (NRM). **(D)** Cumulative incidence of relapse (CIR). **(E)** OS rate among patients who achieved CR with first-line treatment. **(F)** NRM among patients who achieved CR with first-line treatment. **(G)** OS rate comparison between the aGVHD and no-aGVHD subgroups. **(H)** CIR comparison between aGVHD and no-aGVHD subgroups.

The estimated 1-year OS was 84.6% (95% CI: 57.8%–95.7%) (**Figure 4A**), and the estimated 1-year progression-free survival (PFS) probability was 84.6% (95% CI: 57.8%–95.7%) (**Figure 4B**). The 1-year non-relapse mortality (NRM) and cumulative incidence of relapse (CIR) were 7.7% (95% CI: 1.4%–33.3%) each (**Figures 4C, D**). For patients who achieved first complete remission (CR1), the 1-year OS was 90.9% (95% CI: 62.3%–98.4%) (**Figure 4E**) and the 1-year NRM was 9.1% (95% CI: 1.6%–37.7%) (**Figure 4F**). In addition, patients were divided into aGVHD and no-GVHD subgroups. OS did not significantly differ between the aGVHD and no-GVHD groups (**Figure 4G**). The cumulative incidence of relapse also did not significantly differ between the aGVHD and no-GVHD subgroups (**Figure 4H**, p=0.27).

For the 11 patients without CMV reactivation, the median follow-up time was 1011 days (range: 657–1561 days) post-HSCT. Two patients died of severe infection, resulting in a 1-year NRM rate of 7.7% (95% CI: 1.4%–33.3%).

### Sustained expansion and long-term persistence of TCR-T cells *in vivo*

We monitored circulating CMV-TCR-T cells using qPCR at standardized post-infusion time points in all patients with CMV reactivation. These cells exhibited robust *in vivo* expansion and persistence. CMV-TCR-T cells were first detected 6 days (range: 3–7 days) post-infusion. In the 11 patients who sustained a negative CMV status, amplification was significant (median peak: 12,556 copies/μg DNA; range: 5,512–219,686 copies/μg DNA) and persisted for at least 114 days. The median time to reach the peak was 42 days (range: 7–114 days). We further summarized peak TCR copy number and time-to-peak according to corticosteroid exposure at the time of infusion as a descriptive comparison (**Supplementary Table 6**).

Patients 01, 04, and 09 received steroid maintenance for pre-existing aGVHD at the time of infusion; however, the TCR-T cells still expanded efficiently *in vivo*. At the final monitoring point on day 228 post-infusion, TCR copies remained detectable in Patient 09 at 1×10^5^ copies/μg DNA, indicating robust proliferative capacity *in vivo.* All patients exhibited TCR persistence of at least 114 days, including those under immunosuppressive conditions, with some showing persistence for up to 228 days.

In Patient 04, who failed to achieve CMV clearance, TCR-T cells were detected as early as day 3 after infusion and peaked on day 4, but the peak level remained low (1.2 × 10^3^ copies/μg DNA) and persistence was short (12 days only), in contrast to sustained responders, who showed a median peak level of 12,556 copies/μg DNA and persistence for at least 114 days. This patient had pre-existing grade 3 gastrointestinal aGVHD and was receiving methylprednisolone plus basiliximab before infusion, suggesting an immunosuppressive host environment that may have restricted *in vivo* expansion and persistence of the transferred cells. In addition, the patient had tissue-invasive CMV colitis with initially negative peripheral blood CMV DNA, indicating possible compartmentalized gastrointestinal infection in which peripheral blood monitoring may have underestimated the true tissue disease burden. Notably, the infused product for Patient 04 showed no obvious abnormalities in routine characterization, with a phenotype comparable to that of the overall cohort, a cell viability of 87%, and a CD3+/CD8+Tetramer+ transduction efficiency of 30% (**Supplementary Table 4**). Taken together, these findings suggest that treatment failure in Patient 04 was likely multifactorial, involving a high-risk immunosuppressive background, compartmentalized tissue infection, and insufficient *in vivo* expansion/persistence.

In Patient 06, who later experienced CMV recrudescence on day 104 post-HSCT, TCR-T cells were detected in circulation on day 7 after infusion and expanded continuously, peaking on day 63 (1.5 × 10^5^ copies) and remaining readily detectable at day 114. At the last TCR follow-up time point on day 228, peripheral blood TCR copies were no longer detectable. Therefore, based on the available sampling schedule, the exact temporal relationship between CMV recrudescence and loss of detectable peripheral blood TCR copies could not be determined. Moreover, this patient had already developed leukemia relapse on day 95 post-HSCT and subsequently received blinatumomab, both of which may have substantially affected immune reconstitution as well as the persistence and function of transferred TCR-T cells. Accordingly, this case should be interpreted as a descriptive observation rather than evidence of a direct causal relationship between peripheral blood TCR-T persistence and CMV protection. In addition, because endogenous CMV-specific T-cell reconstitution was not directly measured in this study, any inference regarding protection before endogenous immune recovery remains speculative. It should also be noted that the predefined 228-day TCR monitoring window reflects the standardized molecular surveillance schedule of this study rather than the limit of clinical follow-up. CMV-related clinical endpoints continued to be monitored beyond the period of TCR detectability, and no additional CMV-related clinical outcome changes were observed during extended follow-up.

To monitor the clonal frequency of TCRs at different time points post-infusion, immune repertoire sequencing was performed in Patients 01, 07, and 13. In Patient 07, CMV-specific TCRs represented 2.96%, 4.80%, and 4.72% of the repertoire on days 30, 90, and 180, respectively, and became undetectable on day 360, indicating persistence for at least 180 days. In Patients 01 and 13, clonal frequencies were 1.21% and 0.53% on day 30, 3.27% and 0.81% on day 90, and undetectable on day 180, confirming the persistence of CMV-TCR-T cells for at least 90 days. These findings aligned with qPCR results, confirming the long-term *in vivo* persistence of CMV-specific TCR sequences and their role in protective immunity.

Further analysis of TCR clonal diversity initially revealed low clonal diversity in patients post-HSCT, followed by gradual recovery. By day 180, the TCR diversity in Patients 01, 07, and 13 approached normal levels, which was consistent with lymphocyte subset data.

### TCR-T therapy showed promising efficacy relative to the concurrent control cohort

Although randomization was not applied in our study, we evaluated outcomes in the HLA-mismatched, CMV reactivation concurrent control cohort (Concurrent Cohort 2, n=24). Among the 24 patients, 20 received ganciclovir as first-line treatment, whereas the remaining four, who exhibited myelosuppression, received foscarnet as the primary treatment. The median age was 32 years (range: 10–57 years). CMV reactivation occurred at a median of 50 days post-HSCT (range: 32–140 days). Seventeen patients presented with viremia, three with CMV disease, and four with both. Baseline characteristics of all 24 patients are detailed in **Supplementary Table 3**.

The 4-week CR rate in the concurrent control cohort was 11/24 (45.8%), significantly lower than that in the TCR-T cell cohort (12/13, 92.3%, p=0.01). Patients who did not achieve CR were switched to second-line therapy. Four patients (4/24, 16.7%) showed no response. The median time to achieve CR was 32 days (range: 7–49 days), which was 10 days longer than that in the TCR-T cohort (22 days, p<0.01). Median follow-up time of the concurrent control cohort was 568 days (range: 362–721 days). The 1-year OS was 75% (TCR-T cohort: 84.6%, p >0.99) (**Supplementary Figure 1A**), and the 1-year PFS was 83.3% (TCR-T cohort: 84.6%, p >0.99) (**Supplementary Figure 1B**). Two patients died from relapse, and four died from non-relapse causes. The 1-year NRM was 18.2% (TCR-T cohort: 7.7%, p=0.48) (**Supplementary Figure 1C**), and the 1-year CIR was 8.3% (TCR-T cohort: 7.7%, p=0.78) (**Supplementary Figure 1D**).

Treatment-related toxicity occurred in 15 patients (15/24, 62.5%) in the control cohort. Grade ≥3 AEs were reported in five patients (5/24, 20.8%). The most common AEs (all grades, reported as number of events) included nausea and vomiting (n=9), elevated serum creatinine (n=3), neutropenia (n=4), and thrombocytopenia (n=7). One patient required dose adjustment for renal toxicity. In contrast, in the TCR-T cohort, only two cases of grade 1 CRS, both of which resolved spontaneously, were reported. This highlights the favorable safety profile of TCR-T cell therapy.

In addition, descriptive survival analysis was performed on the other two concurrent control cohorts: HLA-matched, no CMV reactivation (Concurrent Cohort 1) and HLA-mismatched, no CMV reactivation (Concurrent Cohort 3). The median follow-up time of Concurrent Cohort 1 was 664 days (range: 450–1414 days), with 1 patient dying from relapse of the primary disease and 1 patient dying from non-relapse-related causes. 1-year OS rate was 81.8% (95% CI: 52.3%–94.9%) (**Supplementary Figure 3A**), and 1-year PFS rate was 90.9% (95% CI: 62.3%–98.4%) (**Supplementary Figure 3B**). 1-year non-relapse mortality (NRM) rate was 9.1% (95% CI: 1.6%–37.7%) (**Supplementary Figure 3C**), and 1-year cumulative incidence of relapse (CIR) was 9.1% (95% CI: 1.6%–37.7%) (**Supplementary Figure 3D**). The median follow-up time of Concurrent Cohort 3 was 559 days (range: 455–755 days), with 1 patient dying from relapse of the primary disease and 2 patients dying from non-relapse-related causes. 1-year OS rate was 85.0% (95% CI: 64.0%–94.8%) (**Supplementary Figure 3A**), and 1-year PFS rate was 85.0% (95% CI: 64.0%–94.8%) (**Supplementary Figure 3B**). 1-year NRM rate was 10.0% (95% CI: 2.8%–30.1%) (**Supplementary Figure 3C**), and 1-year CIR was 5.0% (95% CI: 0.9%–23.6%) (**Supplementary Figure 3D**).

## Discussion

We conducted a single-arm, open-label, phase 2 clinical trial demonstrating that CMV TCR-T cell therapy was significantly effective and well-tolerated as a first-line treatment for CMV reactivation after haplo-HSCT. To our knowledge, this is among the largest prospective clinical studies of CMV-specific TCR-T therapy in this setting.

Our data, based on 13 patients with CMV reactivation after haplo-HSCT, showed that 12 of 13 patients (12/13, 92.3%) achieved CR by week 4. Among the responders, 11 of 12 patients (11/12, 91.7%) maintained sustained CR without additional antiviral treatment. Notably, among the 12 patients who achieved CR, only one (1/12, 8.3%) experienced CMV recrudescence during follow-up. In comparison, a large study of donor-derived CMV-specific CTLs after haploidentical HSCT reported a cumulative complete response rate of 89.5% by week 6 (30). Cross-trial comparisons should be interpreted cautiously; however, our results compare favorably with previously reported outcomes of donor-derived CMV-specific CTLs, rendering them an alternative option for providing long-term antiviral protection. This may be attributed to the adoptive transfer of long-persisting TCR-T cells, whereas CMV-CTLs were exogenously infused. Ultimately, recovering the quantity and function of endogenous (i.e., donor-derived) CTLs is necessary to achieve antiviral immune reconstitution. Nevertheless, TCR-T cells, which are genetically engineered T cells that can undergo clonal expansion *in vivo*, would facilitate their persistence and long-term immune protection.

The application of both TCR-T cell and CTL therapies is limited because they require antigen recognition through MHC molecules (11, 31). Consequently, their effectiveness depends on the patient-specific human leukocyte antigen (HLA) profile, which requires individualized cell modifications. Current clinical trials have focused on HLA-A*02:01, HLA-A*24:02, and HLA-A*11:01, which cover a significant proportion of the population. In our study, we expanded this range to include HLA-A*02:06 and HLA-A*02:07 (32, 33), which are prevalent in East Asian populations, particularly in the Han Chinese population. Expanding the range of HLA types can lead to better overall coverage and provide treatment options for over 50% of patients with CMV infection.

CMV reactivation affects immune cell recovery, particularly that of T-cell populations, post-transplantation, and may lead to a decline in the number of T-cell subsets involved in immune surveillance, further impairing immune reconstitution (34). Additionally, memory T-cell reconstitution is disrupted, compromising long-term immune protection (35). The impact of TCR-T cell therapy on immunosuppression in CMV reactivation post-haplo-HSCT has not been fully described. In our study, the absolute counts of total T, CD4+ T, and CD8+ T cells at 90, 180, and 360 days in the CMV TCR-T group were higher than those in the control group. Notably, CD8+ T cells showed a significant increase at 180 days, likely owing to the infusion of TCR-T cells, potentially accelerating CD8+ T-cell reconstitution. By day 360, immune reconstitution was relatively complete in both the TCR-T and concurrent control groups, with no significant differences observed between the groups. These findings suggest that TCR-T cell therapy may enhance long-term T-cell immune reconstitution, particularly for CD8+ T cells. Our data show that, in the 11 patients who achieved sustained CR, recovery of CD4^+^ T cells to >50/μL occurred after CR had already been achieved, suggesting that early durable viral control was more likely mediated by the infused TCR-T cells than by spontaneous endogenous immune reconstitution. In Patient 04, the only primary non-responder, grade 3 aGVHD developed on day 77 post-HSCT and was treated with corticosteroids (1 mg/kg) plus five doses of basiliximab. The patient received two TCR-T infusions on days 85 and 92 post-transplant, whereas CD4^+^ T-cell recovery to >50/μL was not observed until day 135 post-HSCT, well after both infusions and the protocol-defined response evaluation period. This temporal pattern argues against early endogenous T-cell recovery as the main explanation for treatment failure. Instead, the heavily immunosuppressed host environment may have limited the *in vivo* expansion, persistence, and antiviral activity of the infused TCR-T cells. Nevertheless, given the small sample size and the presence of only one primary non-responder, this observation should be interpreted cautiously, and additional studies are needed to further clarify the mechanisms underlying treatment failure and immune reconstitution (36).

Recipient CMV serostatus is a key determinant of CMV reactivation risk post-transplant. In our study, all patients in both the TCR-T and control cohorts were D+/R+, representing CMV-seropositive recipients who received grafts from CMV-seropositive donors. This donor-recipient CMV serostatus pairing leads to a higher reactivation risk, particularly in CMV-seropositive recipients (R+), due to the absence of donor-transferred CMV-specific immunity (1). In D+/R+ recipients, CMV reactivation is primarily driven by the lack of immune control during reactivation of latent CMV. TCR-T cells, which are engineered to target CMV antigens, provide an effective solution for these high-risk patients (5). Our study suggests that TCR-T therapy can significantly reduce the risk of reactivation in CMV-seropositive recipients by restoring antigen-specific immune responses. The role of D/R serostatus pairing in influencing reactivation rates underscores the need for further studies comparing CMV-seropositive (D+/R+) and CMV-seronegative (D-/R-) subgroups. Such studies could provide more insights into how TCR-T therapy performs across different serostatus profiles and help refine treatment strategies for various patient groups.

Notably, CMV-TCR-T cells demonstrated proliferative capacity even in patients receiving immunosuppressants for GVHD. Previous studies have indicated that steroid therapy poses a significant challenge to adoptive T-cell therapy because glucocorticoids suppress the activation and expansion of transferred T cells (37, 38). However, in this study, corticosteroid treatment did not significantly affect antiviral efficacy. Nevertheless, the single primary highlights an important boundary condition of this approach. This patient had grade 3 gastrointestinal aGVHD requiring methylprednisolone and basiliximab, tissue-invasive CMV colitis with initially PCR-negative peripheral blood, and markedly limited TCR-T expansion/persistence *in vivo*. These features suggest that severe immunosuppressive host conditions and compartmentalized tissue infection may restrict the ability of CMV-TCR-T cells to achieve durable virologic control, even when partial clinical and histopathological improvement is observed. Although the low peak TCR copy number in this patient may be associated with treatment failure, our cohort size and the presence of only one primary non-responder do not allow definition of a reliable predictive threshold. This question should be addressed in larger studies. Three patients received >1 mg/kg/day of methylprednisolone within two weeks post-infusion, and CMV-TCR-T cell expansion was still observed in these cases. A descriptive comparison of peak TCR copy number and time-to-peak according to corticosteroid exposure at the time of infusion is provided in **Supplementary Table 6**. Given the small sample size, this finding should be interpreted as a descriptive observation rather than evidence that corticosteroid exposure does not affect antiviral efficacy.

No cases of ICANS or TCR-T infusion-related GVHD were observed, with only one case of grade 1 CRS that resolved spontaneously within a short period. This safety profile contrasts with that observed in solid tumor TCR-T trials, where higher-grade CRS and ICANS were observed more frequently (39, 40). This outcome may result from the substantially low infusion dose required for efficacy, expansion, and persistence in this setting, in contrast to the higher effective doses typically required for solid tumors (range: 10^8–^10^9^ TCR-T cells/kg) (40–46). The low effective dose of CMV-TCR-T cells also reduces the challenges associated with timely manufacturing. This observation may be partly attributed to the relatively low viral load in our patients at the time of TCR-T cell infusion, which aligns with the favorable safety profile of CMV-CTLs observed in post-transplant CMV infections (30), suggesting that TCR-T cell therapy exhibits favorable tolerability.

In the first-line management of CMV reactivation following HSCT, the safety profile of TCR-T cell therapy must be contextualized within the toxicity framework of conventional antiviral agents. Although maribavir demonstrates reduced toxicity compared with conventional antivirals, clinically relevant adverse events still occur in phase 3 studies. In the AURORA trial, grade 3/4 neutropenia occurred in 16.1% of maribavir-treated patients compared with 50.0% of valganciclovir-treated patients (8, 9). In addition, resistance remains an important concern. In a resistance analysis of the phase 3 SOLSTICE trial, 48% of maribavir nonresponders developed maribavir resistance mutations (47). In contrast, TCR-T cell therapy can help achieve durable virologic control through CMV antigen-specific targeting (such as pp65/IE1) coupled with host immune reconstitution, demonstrating superior tolerability. We also observed no cases of CRS requiring intervention, and most patients maintained sustained viral remission after 1–2 infusions, with recrudescence occurring in 1 of 12 patients who achieved CR (8.3%). This recrudescence rate appears numerically lower than that reported in some maribavir studies, while avoiding cumulative drug toxicity. This risk-benefit profile positions TCR-T therapy as a prospective alternative for HSCT patients with compromised organ function or resistance-prone CMV strains. Another theoretical safety concern in TCR-engineered T-cell therapy is potential mispairing between endogenous and introduced TCR chains, which could theoretically generate off-target reactivity. Dedicated assays specifically evaluating TCR mispairing were not performed in the present study. Therefore, this possibility cannot be completely excluded at the mechanistic level and should be considered a limitation of the current work. Nevertheless, no clinical events suggestive of unexpected TCR-T–related toxicity, such as ICANS, TCR-T–associated GVHD, or secondary malignancies, were observed in this cohort. Future translational studies incorporating more comprehensive molecular and functional analyses will be required to further evaluate this issue.

The small sample size is a key limitation that may affect the robustness and the generalizability of our findings. A large-scale, multicenter study is required to validate these results.

A further methodological consideration is that the study used a uniform prespecified CMV DNA threshold to trigger TCR-T infusion across all patients, rather than a risk-adapted threshold strategy. Although this approach improved protocol consistency in this exploratory phase 2 trial, it may appear relatively conservative for patients who had developed GVHD before TCR-T therapy and were still receiving tapering steroids or other ongoing immunosuppressive therapy at the time of CMV reactivation. Notably, however, none of the five high-risk treated patients in this category had detectable but sub-threshold viremia prior to meeting the qualifying event, suggesting that the use of a uniform threshold did not result in a documented delay attributable to low-level antecedent viremia in these cases. Future studies should nevertheless evaluate whether earlier intervention at lower-level viremia may be preferable in such high-risk subgroups.

In addition, although the highest dose level was selected for Phase II because of its faster apparent onset of efficacy in Phase I, future prospective studies should evaluate whether lower-dose or risk-adapted dosing strategies can preserve efficacy while reducing manufacturing burden and improving scalability.

Relative to conventional antiviral regimens, TCR-T therapy was associated with an observed higher 4-week CR rate(92.3% vs. 45.8% in controls), faster viral clearance, a lower recrudescence rate (8.3%), and no grade ≥3 treatment-related toxicities, which may help reduce indirect costs associated with prolonged hospitalization, organ toxicity management, salvage antiviral therapy, and secondary infections. By accelerating immune reconstitution and abrogating antiviral drug resistance, this therapy optimizes peri-transplant care pathways and cuts long-term healthcare resource utilization. Future large-scale studies incorporating formal health economic evaluations, including cost–effectiveness and cost–utility analyses, are warranted to quantify its full-cycle economic value and optimize risk-stratified infusion strategies, supporting further prospective validation of this approach in routine HSCT clinical management.

In conclusion, CMV-specific TCR-T cells are highly effective and well tolerated as a first-line preemptive treatment for CMV reactivation after haplo-HSCT, with no observed TCR-specific toxicities, and may be particularly attractive in settings where conventional antivirals are limited by hematologic or renal toxicity. These preliminary findings support CMV-specific TCR-T cell therapy as a promising therapeutic approach for this high-risk population and justify further clinical investigation.

## Data Availability

The original contributions presented in the study are included in the article/**Supplementary Material**. Further inquiries can be directed to the corresponding authors.
