# Supplementary material for: CMV-specific T-cell receptor-engineered T-cell therapy as first-line treatment for CMV reactivation after haploidentical hematopoietic stem cell transplantation: a phase 2 trial
Source: Front Immunol. 2026 May 14;17:1820399. doi: 10.3389/fimmu.2026.1820399 (PMC13216466; doi:10.3389/fimmu.2026.1820399)
Supplement: Supplementary file 1 [file DataSheet1.pdf]

TCR-T cell preparation

1. Timing and Efforts for TCR-T Cell Preparation

Manufacturing timeline: All TCR-T cells were produced in a clinical-grade GMP cell manufacturing laboratory with a median preparation time of 13 days (range: 10–13 days). This cycle covers the entire process from peripheral blood mononuclear cell (PBMC) collection, viral vector construction, lentivirus-mediated TCR gene transduction, in vitro directed expansion to product release testing, which is significantly shorter than the manufacturing cycle of conventional CMV-specific CTLs.

Manufacturing process and efforts: A standardized GMP operational workflow was adopted, with core steps including: ① Collection of donor PBMCs and isolation by density gradient centrifugation; ② Screening, cloning of CMV pp65/IE1 antigen-specific TCR sequences and construction of lentiviral vectors; ③ Lentiviral transduction of CD3+ T cells; ④ In vitro expansion supported by anti-CD3/CD28 immunomagnetic bead activation combined with cytokines; ⑤ Cell washing, concentration, formulation preparation, and quality testing. The entire process strictly complies with the "Good Manufacturing Practices for Cell Therapy Products".

2. Source of PBMCs and Storage of TCR-T Cells

Source of PBMCs: All PBMCs were derived from the same hematopoietic stem cell transplant donor matched with the patient's HLA-A genotype. Donors were required to carry at least one of the HLA-A alleles (HLA-A*02:01, 11:01, 24:02, 02:06, or 02:07) to ensure antigen recognition compatibility of TCR-T cells, which is consistent with the prespecified donor eligibility criteria (⩾3/6 HLA matching with recipients, healthy donors).

Storage method: After passing quality inspection, the prepared TCR-T cell formulations were aliquoted in cryopreservation medium containing 10% dimethyl sulfoxide (DMSO) and 40% fetal bovine serum, and stored in liquid nitrogen at -196℃. Prior to clinical use, the formulations were rapidly thawed in a 37℃ water bath and infused immediately. The cold chain environment and contamination risks were strictly controlled throughout the process.

3. Quality Control Standards for TCR-T Cells

Key quality indicators have been detailed in the study's methodology section and supplementary materials, with critical test results as follows:

Transduction efficiency: Using the proportion of CD8+Tetramer+ cells as the core indicator, the

median transduction efficiency was 28.41% (range: 11.64% – 77.56%) with a prespecified quality control threshold of ≥1%. All products met the release criteria.

Cell viability: Flow cytometry detected that cell viability was ≥80% before infusion (the viability of the product used in the clinical application case reached 87%), ensuring the biological activity of the infused cells.

Safety testing: Including sterility testing (no bacterial or fungal growth), endotoxin testing (<5 EU/mL), and mycoplasma testing (negative). All products passed the release testing without safety hazards.

Cytotoxicity Validation: We have performed comprehensive in vitro cytotoxicity assays to validate the HLA- and antigen-specific activity of the CMV-specific TCR-T cells. For each TCR construct, we measured cytotoxicity against HLA-matched target cells loaded with the corresponding CMV peptide and HLA-mismatched controls at multiple effector-to-target (E:T) ratios (12:1, 6:1, and 3:1). The results show that CMV-specific TCR-T cells preferentially lysed HLA-matched target cells in a dose-dependent manner, with cytotoxicity increasing from approximately (E:T 3:1: 5%–22%) to (E:T 6:1: 28%–69%) and further to (E:T 12:1: 43%–88%). In contrast, HLA-mismatched target cells exhibited only low-level background lysis, comparable to NC cells (range: 4%-27%) **(Supplementary Figure 2)**. These data confirm the **HLA-restricted and antigen-specific cytotoxic activity** of all TCR-T constructs. Cell phenotype: The product was dominated by functional T cells, with a median CD3+ cell proportion of 99.63% and a CD3+CD8+ cell proportion of 54.4%, ensuring the targeted killing efficacy of the formulation.

4. Description of Antigen-Specific Effective Dose

To accurately reflect the actual effective cell exposure received by each patient, we have added the "estimated infused CD8+Tetramer+ cell dose" for each patient in the supplementary materials. This dose is calculated based on the product viability, transduction efficiency, and total infused dose (5×10^5 cells/kg), which complements the limitation of only reporting the total cell dose and enables more accurate interpretation of the dose-effect relationship.

**Inclusion Criteria for CMV TCR-T Cell Trial Participants**	1. Age 14–75 years at screening, regardless of sex.<br>2. Diagnosed with hematologic or lymphoid malignancies, eligible for or having undergone allogeneic hematopoietic stem cell transplantation (allo-HSCT), and with post-transplant CMV reactivation.<br>3. At least 6/12 HLA match with the transplant donor, and genotype must match at least one of the following HLA-A types: HLA-A02:01, HLA-A11:01, HLA-A24:02, HLA-A02:06, or HLA-A*02:07.<br>4. CMV-DNA positive at screening.<br>5. Karnofsky performance score (≥16 years) ≥70 or Lansky score (<16 years) ≥50.<br>6. Voluntary participation in the clinical study; the participant or legal guardian fully understands the study and signs the informed consent form (ICF).
**Exclusion Criteria for CMV TCR-T Cell Trial Participants**	1. History of allergy to any component of the cell product.<br>2. Uncontrolled graft-versus-host disease (GVHD) prior to cell infusion.<br>3. History of other malignancies within 5 years before screening, except carcinoma in situ (e.g., cervical, bladder, or breast carcinoma in situ) or non-melanoma skin cancer.<br>4. Active or uncontrolled relapse of the primary malignancy (except EBV-positive post-transplant lymphoproliferative disease or lymphoma).<br>5. Positive for any of the following: HBsAg, HBeAg, HBe-Ab, HBc-Ab, HBV-DNA above normal limits; anti-HCV, HCV-RNA above normal limits; HIV-Ab; TP-Ab; CMV-DNA >1000 copies/mL, CMV-IgM positive; EBV-DNA >1000 copies/mL, EBV-IgM positive or above normal limits.<br>6. Planned receipt of new cellular therapies within 4 weeks after cell infusion.<br>7. Severe heart failure (NYHA class IV, or left ventricular ejection fraction <50%).<br>8. Pregnancy or lactation; or men and women of reproductive potential who refuse to use contraception during the study period and for 24 weeks after cell infusion.<br>9. Receipt of live attenuated vaccines within 1 month prior to screening or expected need for such vaccines during the study period.<br>10. Any condition that, in the investigator's judgment, makes the participant unsuitable for participation in the study.
**Inclusion Criteria for Transplant Donors**	1. Meets the clinical allo-HSCT donor eligibility criteria.<br>2. Age ≥8 years and ≤70 years, regardless of sex.<br>3. The donor or legal guardian fully understands the study and signs the informed consent form.<br>4. At least 6/12 HLA match with the recipient, and genotype must match at least one of the following HLA-A types: HLA-A02:01, HLA-A11:01, HLA-A24:02, HLA-A02:06, or HLA-A*02:07.

	5. Lymphocyte count ⩾0.8 × 10^9/L.<br>6. Adequate venous access for leukapheresis or blood collection, with no contraindications to blood cell separation.
**Exclusion Criteria for Transplant Donors**	1. Pregnancy.<br>2. Positive for HBsAg, HBeAg, HBe-Ab, HBc-Ab, HBV-DNA above normal limits; anti-HCV, HCV-RNA above normal limits; HIV-Ab; TP-Ab; CMV-DNA, CMV-IgM, EBV-DNA, EBV-IgM positive or above normal limits.<br>3. Uncontrolled active infection.<br>4. Use of corticosteroids within 1 week prior to PBMC collection, except for inhaled or topical corticosteroids and physiological replacement therapy for adrenal insufficiency.<br>5. Any condition that, in the investigator's judgment, makes the donor unsuitable for donation.

Supplementary Table 1. Inclusion and exclusion criteria for patients and TCR-T cell donors

	HLA-Matched Group (n=25)	HLA-Mismatched Group (n=44)	*P*
**Age, median (range), years**	39 (18-67)	40 (18-66)	0.664
**Sex, No. (%)**			
Male	17 (68.0)	36 (81.8)	0.191
**ECOG pre-SCT, median (range)**	1 (0-2)	1 (0-2)	0.457
**Donor-patient sex match, No. (%)**			0.612
Male-male	12 (48.0)	21 (47.7)	
Male-female	4 (16.0)	4 (9.1)	
Female- female	3 (12.0)	4 (9.1)	
Female- male	5 (20.0)	15 (34.1)	
**ABO match, No. (%)**			0.782
Matched	7 (28.0)	9 (20.5)	
Minor mismatched	6 (24.0)	15 (34.1)	
Major mismatched	10 (40.0)	15 (34.1)	
Different	2 (8.0)	5 (11.4)	
**HLA compatibility, No. (%)**			0.644
1-Locus mismatch	1 (4.0)	2 (4.5)	
2-Locus mismatch	2 (8.0)	2 (4.5)	
3-Locus mismatch	3 (12.0)	2 (4.5)	
4-Locus mismatch	4 (16.0)	5 (11.4)	
5-Locus mismatch	6 (24.0)	18 (40.9)	
6-Locus mismatch	9 (36.0)	15 (34.1)	
**Underlying Hematologic Malignancy No. (%)**			
AML	18 (72.0)	26 (59.1)	0.322
ALL	4 (16.0)	12 (27.3)	
MDS	2 (8.0)	6 (13.6)	
NHL、ALL	1 (4.0)	0	
**Disease Status at Transplant No. (%)**			
CR1	17 (68.0)	35 (79.5)	0.472
CR2	7 (28.0)	7 (15.9)	
No Remission	1 (4.0)	2 (4.5)	
**CMV reactivation, No. (%)**	14 (56.0)	24 (54.5)	0.838
Viremia	11 (11/13, 84.6)	18 (18/24, 75.0)	
Disease	3 (3/13, 23.1)	6 (6/24, 25.0)	
Colitis	2 (2/3, 66.7)	5 (5/6, 83.3)	
Retinitis	0	1 (1/6, 16.7)	
Pneumonia	1 (1/3, 33.3)	0	

Supplementary Table 2. Baseline Demographic, Clinical, and Transplantation Characteristics of the TCR-T and Concurrent Control Cohorts

ECOG, eastern cooperative oncology group; HLA, human leukocyte antigen; SCT, stem cell transplantation; CMV, Cytomegalovirus.

Patient ID	Diagnosis	Donor	D/R CMV serostatus	Basic conditioning regimen	Day of CMV reactivation	Time to neutrophil engraftment	Time to platelet engraftment	Time to CD4^{+} T cells >50/μL	CMV reactivation symptom
01	AML	Sibling	D+/R+	BU/CY+ ATG	33	10	15	81	Viremia
02	AML	Father	D+/R+	BU/CY+ ATG	35	19	15	65	Colitis
03	AML	Father	D+/R+	BU/CY+ ATG	56	9	12	121	Viremia
04	AML	Son	D+/R+	BU/CY+ ATG	36	11	10	240	Viremia
05	ALL	Father	D+/R+	BU/CY+ ATG	71	14	15	76	Colitis
06	ALL	Father	D+/R+	BU/CY+ ATG	38	14	29	95	Viremia
07	ALL	Sibling	D+/R+	BU/CY+ ATG	49	12	10	134	Viremia
08	ALL	Son	D+/R+	FB+ATG	43	15	17	78	Viremia
09	AML	Son	D+/R+	BU/CY+ ATG	32	18	11	43	Viremia
10	AML	Father	D+/R+	BU/CY+ ATG	54	11	12	123	Viremia
11	ALL	Sibling	D+/R+	BU/CY+ ATG	57	13	13	219	Viremia
12	AML	Sibling	D+/R+	BU/CY+ ATG	53	15	15	225	Viremia
13	AML	Mother	D+/R+	BU/CY+ ATG	37	12	13	88	Viremia
14	MDS	Sibling	D+/R+	BU/CY+ ATG	140	12	19	103	Colitis
15	AML	Mother	D+/R+	BU/CY+ ATG	44	16	21	178	Viremia
16	AML	Sibling	D+/R+	BU/CY+ ATG	46	9	13	143	Retinitis
17	ALL	Son	D+/R+	FB+ATG	56	15	9	82	Viremia
18	AML	Sibling	D+/R+	BU/CY+ ATG	109	15	15	150	Viremia
19	AML	Sibling	D+/R+	BU/CY+ ATG	93	18	Pre-engraftment death	Pre-engraftment death	Viremia
20	ALL	Daughter	D+/R+	FB+ATG	37	12	12	54	Colitis
21	MDS	Sibling	D+/R+	BU/CY+ ATG	66	17	23	98	Viremia
22	MDS	Son	D+/R+	BU/CY+ ATG	50	15	20	67	Viremia
23	ALL	Daughter	D+/R+	BU/CY+ ATG	68	16	18	87	Viremia
24	AML	Father	D+/R+	BU/CY+ ATG	44	11	16	90	Colitis

Supplementary Table 3. Baseline characteristics of the concurrent control cohort

Patient ID	Cell phenotype (%)				
	CD3+	CD3+CD4+	CD3+C8+	CD3-CD19+	CD3-CD56+/CD16+
01	99.57	60.42	32.19	0	0
02	100	28.53	65.2	0	0
03	96.71	48.4	47.11	1.97	1.02
04	99.91	24.16	68.09	0	0.01
05	99.72	35.38	61.74	0	0
06	100	49.56	50.41	0	0
07	99.99	35.59	63.58	0	0
08	94.5	38.23	54.96	4.29	1.78
09	99.86	70.42	25.98	0	0
10	97.97	49.81	47.42	0	1.72
11	96.93	19.99	71.33	0.87	2.66
12	95.72	29.38	63.72	0.98	2.89
13	99.88	28.13	64.39	0	0
Median	99.63 (94.27–100)	44.97 (19.84–70.96)	54.4 (24.21–76.8)	0.02 (0–5.43)	1.02 (0–2.89)

Supplementary Table 4: Phenotype and release-related characteristics of TCR-T cell products

TCR construct ID	Targeted CMV epitope sequence	CMV antigen source	HLA restriction
TCR-01	NLVPMVATV	pp65	HLA-A*02:01
TCR-02	QYDPVAALF	pp65	HLA-A*24:02
TCR-03	TPRVTGGGAM	pp65	HLA-A*11:01
TCR-04	VTEHDTLLY	IE1	HLA-A*02:06
TCR-05	SLFNVATSV	pp65	HLA-A*02:07

Supplementary Table 5. CMV-specific TCR constructs, targeted epitopes, and HLA restrictions.

Patient ID	Corticosteroid exposure at infusion	Peak TCR copy number (copies/µg genomic DNA)	Time-to-peak (days post-first TCR-T infusion)
01	No	10367	28
02	No	1034	21
03	No	35547	14
04	Yes (1 mg/kg/day methylprednisolone)	1034	21
05	No	16576	114
06	Yes (1.5 mg/kg/day methylprednisolone)	104664	70
07	No	67006	28
08	No	5882	14
09	Yes (0.5 mg/kg/day methylprednisolone)	219686	114
10	No	11228	14
11	No	13276	28
12	Yes (1.2 mg/kg/day methylprednisolone)	81475	70
13	No	33132	7

Supplementary Table 6. Descriptive comparison of peak TCR-T copy number and time-to-peak according to corticosteroid exposure at the time of infusion.

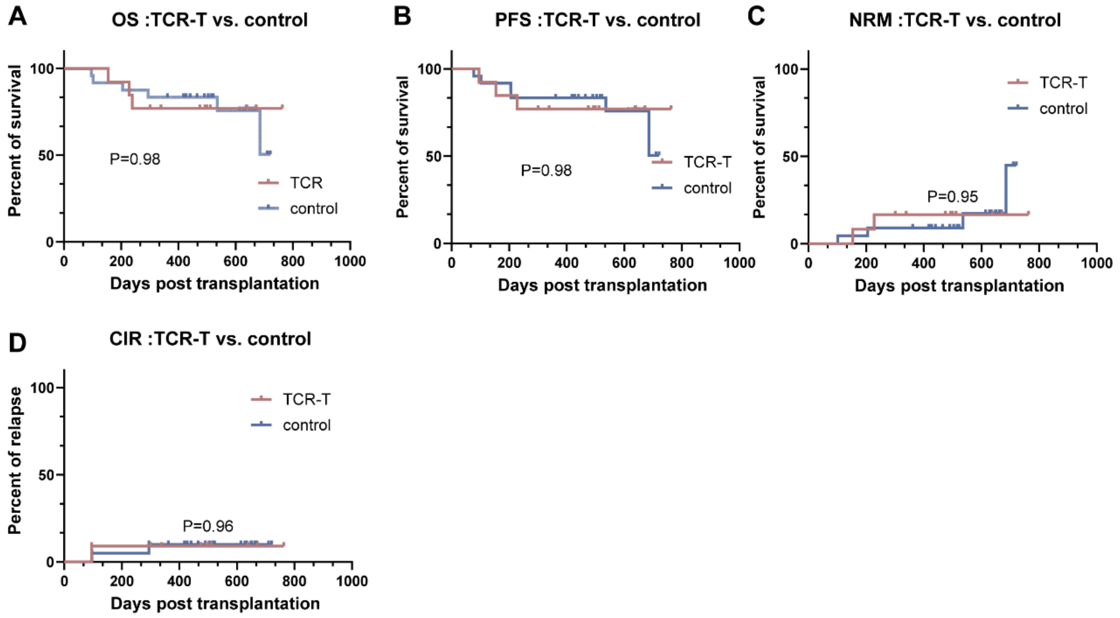

Supplementary Figure 1. Long-term response and survival between the TCR-T group and the concurrent control cohort 2.

(A) Overall survival (OS) rate. (B) Progression-free survival (PFS). (C) Non-relapse mortality (NRM). (D) Cumulative incidence of relapse (CIR).

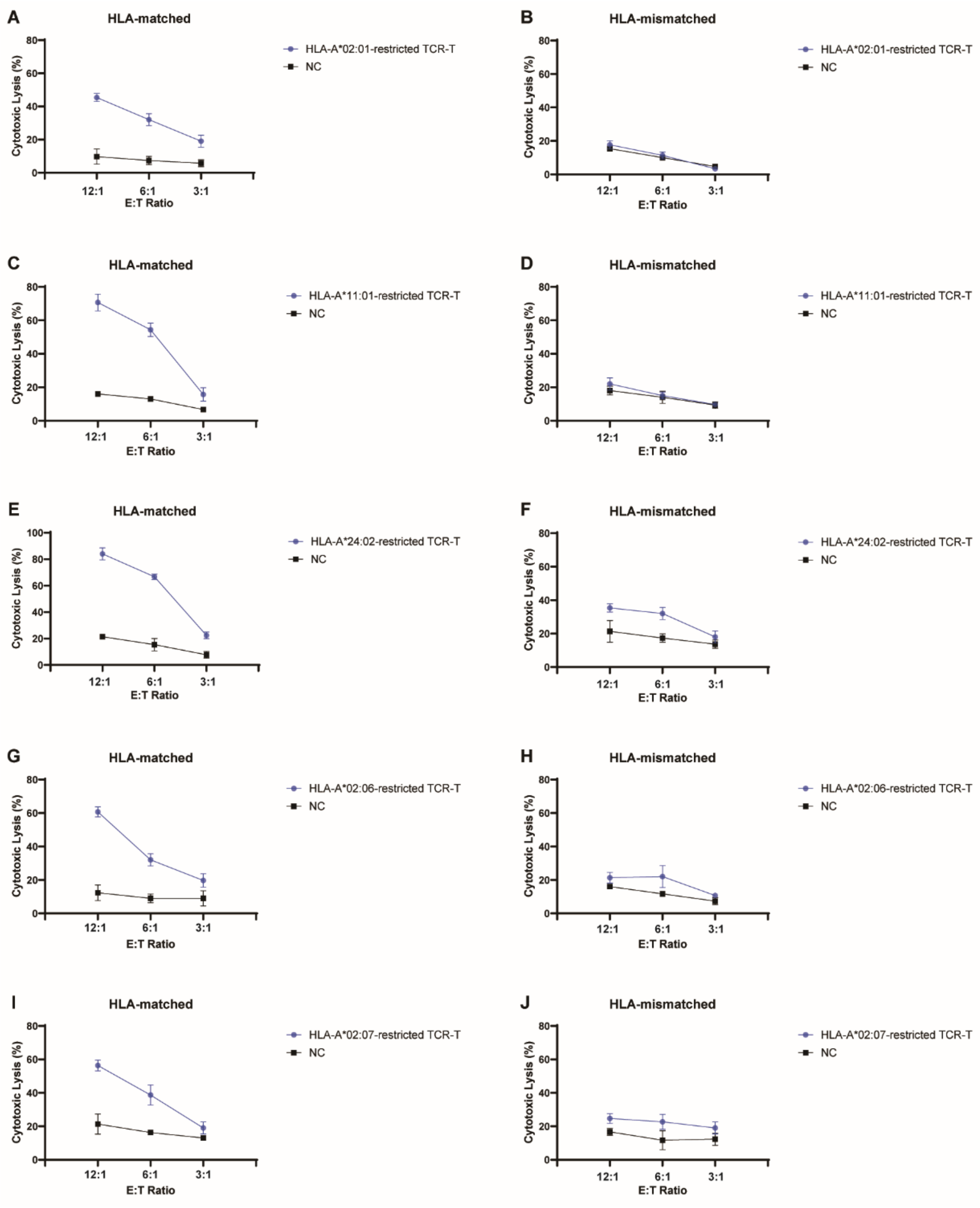

Supplementary Figure 2. HLA-restricted antigen-specific cytotoxicity of CMV-specific TCR-T cells in vitro.

The cytotoxic activity of CMV-specific TCR-T cells was assessed against target cells presenting the corresponding CMV peptide at E:T ratios of 12:1, 6:1, and 3:1. (A,B) HLA-A*02:01-restricted TCR-T cells; (C,D) HLA-A*11:01-restricted TCR-T cells; E,F: HLA-A*24:02-restricted TCR-T cells; (G,H) HLA-A*02:06-restricted TCR-T cells; (I,J) HLA-A*02:07-restricted TCR-T cells. A, C, E, G, and I represent HLA-matched target cells; B, D, F, H, and J represent HLA-mismatched target cells. NC, negative control T cells. CMV-specific TCR-T cells preferentially lysed HLA-matched target cells, confirming HLA-restricted and antigen-specific cytotoxic activity in vitro.

Data are shown as mean ± SD.

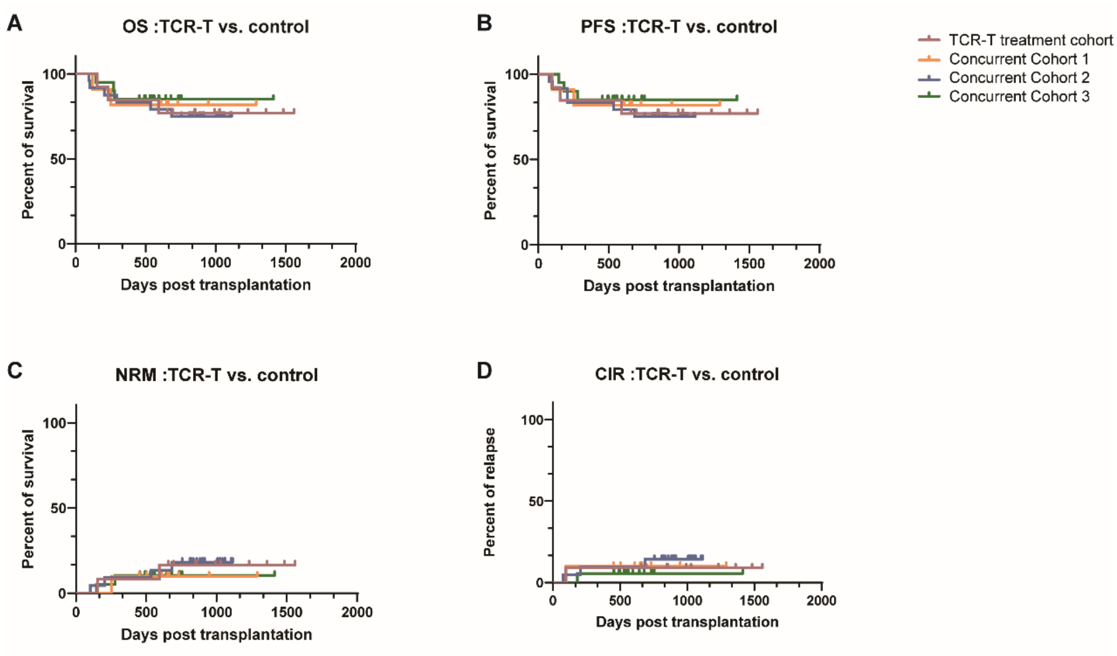

Supplementary Figure 3. Long-term survival outcomes of the TCR-T treatment cohort and three concurrent control cohorts following allogeneic hematopoietic stem cell transplantation. Panels show Kaplan–Meier estimates of (A) overall survival (OS), (B) progression-free survival (PFS), and cumulative incidence estimates of (C) non-relapse mortality (NRM) and (D) cumulative incidence of relapse (CIR) in four patient cohorts. TCR-T treatment cohort: Patients with CMV reactivation who received TCR-T cell therapy (the study cohort of the clinical trial). Concurrent Cohort 1: HLA-matched patients eligible for TCR-T therapy who did not experience CMV reactivation. Concurrent Cohort 2: HLA-mismatched patients with CMV reactivation who received standard antiviral therapy (primary concurrent control for short-term efficacy comparisons). Concurrent Cohort 3: HLA-mismatched patients without CMV reactivation.
